# Analysis of Ovarian Injury Associated With COVID-19 Disease in Reproductive-Aged Women in Wuhan, China: An Observational Study

**DOI:** 10.3389/fmed.2021.635255

**Published:** 2021-03-19

**Authors:** Ting Ding, Tian Wang, Jinjin Zhang, Pengfei Cui, Zhe Chen, Su Zhou, Suzhen Yuan, Wenqing Ma, Minli Zhang, Yueguang Rong, Jiang Chang, Xiaoping Miao, Xiangyi Ma, Shixuan Wang

**Affiliations:** ^1^Department of Obstetrics and Gynecology, National Clinical Research Center for Obstetrical and Gynecological Diseases, Tongji Medical College, Tongji Hospital, Huazhong University of Science and Technology, Wuhan, China; ^2^Department of Pathogen Biology, School of Basic Medicine, Huazhong University of Science and Technology, Wuhan, China; ^3^Department of Epidemiology and Biostatistics, Key Laboratory for Environment and Health, School of Public Health, Tongji Medical College, Huazhong University of Sciences and Technology, Wuhan, China

**Keywords:** COVID-19, infectious disease, ovarian injury, ovarian reserve, sex hormones, reproductive health, observational study

## Abstract

**Objective:** This study was intended to investigate the relationship between COVID-19 disease and ovarian function in reproductive-aged women.

**Methods:** Female COVID-19 patients of reproductive age were recruited between January 28 and March 8, 2020 from Tongji Hospital in Wuhan. Their baseline and clinical characteristics, as well as menstrual conditions, were recorded. Differentials in ovarian reserve markers and sex hormones (including anti-Müllerian hormone [AMH], follicle-stimulating hormone [FSH], the ratio of FSH to luteinizing hormone [LH], estradiol [E2], progesterone [P], testosterone [T], and prolactin [PRL] were compared to those of healthy women who were randomly selected and individually matched for age, region, and menstrual status. Uni- and multi-variable hierarchical linear regression analyses were performed to identify risk factors associated with ovarian function in COVID-19 women.

**Results:** Seventy eight patients agreed to be tested for serum hormone, of whom 17 (21.79%) were diagnosed as the severe group and 39 (50%) were in the basal level group. Menstrual status (*P* = 0.55), menstrual volumes (*P* = 0.066), phase of menstrual cycle (*P* = 0.58), and dysmenorrhea history (*P* = 0.12) were similar without significant differences between non-severe and severe COVID-19 women. Significant lower serum AMH level/proportion (0.19/0.28 vs. 1.12 ng/ml, *P* = 0.003/0.027; AMH ≤ 1.1 ng/ml: 75/70.4 vs. 49.7%, *P* = 0.009/0.004), higher serum T (0.38/0.39 vs. 0.22 ng/ml, *P* < 0.001/0.001) and PRL (25.43/24.10 vs. 12.12 ng/ml, *P* < 0.001/0.001) levels were observed in basal level and the all-COVID-19 group compared with healthy age-matched control. When adjusted for age, menstrual status and parity variations in multivariate hierarchical linear regression analysis, COVID-19 disease was significantly associated with serum AMH (β = −0.191; 95% CI: −1.177–0.327; *P* = 0.001), T (β = 0.411; 95% CI: 11.154–22.709; *P* < 0.001), and PRL (β = 0.497; 95% CI: 10.787–20.266; *P* < 0.001), suggesting an independent risk factor for ovarian function, which accounted for 3.2% of the decline in AMH, 14.3% of the increase in T, and 20.7% of the increase in PRL.

**Conclusion:** Ovarian injury, including declined ovarian reserve and reproductive endocrine disorder, can be observed in women with COVID-19. More attention should be paid to their ovarian function under this pandemic, especially regarding reproductive-aged women.

**Clinical Trial Number:** ChiCTR2000030015.

## Introduction

In December 2019, COVID-19 (caused by the SAR-CoV-2 virus) broke out in Wuhan, China, and rapidly spread across the world. As of Nov 22, 2020, the novel virus had infected over 57.6 million people, caused ~1.4 million deaths in 220 countries (1), and was identified as a pandemic. Unfortunately, about 3.6% patients shadily died of multiple organ failure, acute respiratory distress syndrome, shock, heart, or renal failure. The majority of those infected are still alive, although with organ damage, e.g., in the reproductive system, especially in those reproductive age women (2) who would likely have sought to become pregnant without concern for related gynecological complications (3). While respiratory, cardiac, ocular, urologic, and neurologic symptoms of COVID-19 have been reported, the ovarian implications of SAR-CoV-2 infection are unknown and have rarely been studied. Besides the self-reported menstrual cycle changes, ovarian reserve markers and reproductive hormones, including anti-Müllerian hormone (AMH), follicle-stimulating hormone (FSH), the ratio of FSH to luteinizing hormone (LH), estradiol (E2), progesterone (P), testosterone (T), and prolactin (PRL), should be an effective way to reflect the ovarian function (4) and possible ovarian injury associated with the COVID-19 diseases. However, these tests were seldom to be performed and whether COVID-19 might affect ovarian function is reported on only a limited scale. More concern has emerged regarding the effects of the novel coronavirus on pregnancy outcomes, vertical transmission (5, 6), sexual transmission (7), and the male reproductive system (8). How COVID-19 disease- state and recovery affect the ovary, and the consequences to a female's menstrual cycle, reproduction potential and endocrine function, remain unknown, and urgently need to be studied.

COVID-19 is not only capable of causing respiratory symptoms. It may also cause damage beyond the respiratory system, e.g., to the nervous and immune systems (9, 10) or liver (11, 12) and, according to limited reports, to the reproductive system (13), mainly in males (8). Attachment to the Angiotensin Converting Enzyme 2 (*ACE2*) via the viral spike (S) protein is the primary mechanism for coronavirus entry into the cell; the cellular serine protease (*TMPRSS2*) is employed for S protein priming (14). It is conceivable that infection with the virus could have an impact on reproductive function if these genes were expressed in cells of the female reproductive system. Although there are many more *ACE2* receptors present in the male reproductive system than in the female's, gonadotropin-dependent expression of *ACE2* has been reported in human ovaries (15–17). Notably, *ACE2* mRNA transcripts could be detected in ovaries from reproductive-aged women to postmenopausal women (18). In animals, *ACE2* presents in stroma and granulosa cells as well as oocytes in immature rat ovaries. And *ACE2* also exists in bovine theca cells and granulosa cells (19). In addition, based on single cell RNA sequencing data, a subpopulation of oocytes was found to co-express *ACE2* and *TMPRSS2* in non-human primate ovary, although absent from ovarian somatic cells. All of the foregoing findings suggest that the ovary might be attacked by SARS-CoV-2 through *ACE2*, although related studies are still lacking. Further studies are needed to assess the possible impacts of SARS-CoV-2 infection on ovarian function, which is responsible for female fertility and reproductive endocrine function.

Until now, most COVID-19 studies concerning the ovary have been literature reviews based on previous research or publicly available databases. This study provides original clinical evidence concerning the alteration of ovarian reserve and sex-related hormones in patients affected by COVID-19.

## Materials and Methods

### Participants

Between January 28 and March 8, 2020, we performed an observational, single-center study involving a case group of 78 female patients with COVID-19 who were of reproductive age and who were younger than 50, excluding any patients with ovarian diseases or ovarian surgery history and those who denied our request for blood collection. These patients were from two branches of Tongji Hospital (Sino-French New City Branch and Optical Valley Branch). None were pregnant or had taken oral or transdermal estrogen-containing products such as contraceptives or menopausal hormone treatments. Oral informed consent was obtained from each enrolled patient instead of written consent due to the rapid emergence of this disease. Moreover, written consent might be a potential source of infection. Since no uninfected healthy women were hospitalized at the same period, the control group were recruited from the population that had previously received reproductive function evaluation in Tongji hospital; they were without hormone/radiotherapy/chemotherapy in the past 6 months. One fifty one age-matched healthy women were randomly selected and matched individually for region, menstrual cycle, and according to 1:2 ratios as case: control. The data of their sex hormones were collected on days 2–5 of the menstrual cycle or on any day if amenorrhea existed for more than 3 months.

### Data Collection

Recorded information included exposure history, clinical symptoms, medical history, menstruation information in the last 3 months, reproductive history, and laboratory findings. In their medical histories, comorbidities refer to coexisting chronic diseases including hypertension, diabetes, cardiovascular disease etc. Benign gynecological disease included vaginitis, pelvic inflammatory disease, fibroids, etc. Gynecological surgery history included cesarean, artificial abortion, tubal surgery etc.

Laboratory tests included a complete blood count, erythrocyte sedimentation rate (ESR), C-reactive protein (CRP), and cytokines related to immunity and inflammation. All laboratory testing was performed according to each patient's clinical care needs. After laboratory tests required for patients' routine medical purposes were completed, the residual serum samples were collected for sex hormone profile analysis immediately after its collection.

### AMH and Sex Hormone Detection

Serum concentrations of AMH were measured using the Elecsys AMH kit (Roche, Inc, Basel, Switzerl). Serum FSH, LH, E2, P, T, and PRL levels were measured using a chemoluminescence-based immunometric assay on a UniCelDxI 800 immunoassay system (Beckman Coulter, Inc, California, USA). All of the samples were measured in the same laboratory in Tongji hospital. The intra- and inter-assay coefficients of variation were all below 15%. The lowest amount of AMH that could be detected with a 95% probability in a sample was 0.01 ng/mL.

### Definition

A confirmed case of COVID-19 was defined as a positive result with a real-time reverse-transcriptase–polymerase-chain-reaction (RT-PCR) assay of throat swab specimens (20). Severe SARS-CoV-2 infection was defined according to the American Thoracic Society guideline for community-acquired pneumonia (21) and the Guan et al. (22) study of COVID-19 on admission.

Since blood was collected regardless of the status of menstrual cycle in women in the COVID-19 group, those in the basal level group were chosen for further analysis. The basal level group was defined according to the days after initiation of menses, when blood was collected at menstrual cycle day 1–5 or after amenorrhea of more than 3 months.

### Statistical Analysis

Continuous variables were expressed as medians and interquartile ranges (IQR) as appropriate. Categorical variables were summarized as the counts and percentages (%) in each category. Mann-Whitney U tests (non-parametric) were applied to continuous variables, while chi-square tests and Fisher's exact tests were used for categorical variables as appropriate. Univariable and multivariable hierarchical linear regression analyses were used to test the moderating effects between serum AMH/T/PRL and the clinical characteristics of all the COVID-19 and healthy women. For AMH, in step 1, baseline variables which were associated with AMH in univariable analyses were entered as control variables. In step 2, menstrual status was entered. In step 3, COVID-19 disease was entered. For T/PRL, in step 1, dysmenorrhea or parity were entered as control variables and COVID-19 disease was entered in step 2. Standardized regression coefficient (β), R^2^, R^2^ changes (ΔR^2^), and F value for each step were provided. Pearson correlation analyses were used with laboratory characteristics and serum AMH/T/PRL levels. All analyses were conducted with SPSS software version 19.0. Statistical significance was defined as *P-*values of < 0.05.

## Results

### Baseline and Clinical Characteristics of the Female COVID-19 Patients

The baseline and clinical characteristics of the 78 women infected with COVID-19 who agreed to be tested for serum hormone are shown in Table 1. The median age was 43.5 years (IQR, 36.8–47.0); 17/78 (21.79%) was diagnosed as severe group. They had a median BMI of 22.7 (20.15–25.18). Thirty-six (36) of 75 (48.0%) patients described a recent mental disorder such as anxiety, depression, or insomnia, while 68 of 78 had a history of exposure to other fever/pneumonia patients or the Huanan seafood market; 71 of 78 lived in Wuhan. The median menarche age was 13 years (IQR, 12–13); 21.79% (17/78) patients had one or more comorbidities, 12.00% (9/75) had a history of one or more benign gynecological diseases and 36.00% (27/75) had a history of gynecological surgery. There were no significant differences of ovarian reserve markers and sex hormone levels between COVID-19 women with and without comorbidities, benign gynecological disease and gynecological surgery history, respectively (Supplementary Table 1), suggesting that our study revealed no obvious impacts of medical history on ovarian function. At admission, most common symptoms were fever (58, 74.36%), cough (28, 35.90%), and dyspnea (8, 10.26%). 60.81% (45/74) patients received antiviral treatment including arbidol and oseltamivir, and 59.46% (44/74) patients received antibiotic treatment. None received cortisol therapy before the hormone test.

**Table 1:** Baseline and clinical characteristics of the female patients with COVID-19.

		**Female COVID-19 patients**
**Characteristic**		
Age, years		
Median (IQR)		43.50 (36.75–47.00)
Distribution, *n/N* (%)		
10–30		5/78 (6.41)
31–40		23/78 (29.49)
41–50		50/78 (64.10)
Severe group, *n/N* (%)		17/78 (21.79)
Body mass index (BMI), median (IQR)		22.7 (20.15–25.18)
Recent mental disorder, *n/N* (%)		36/75 (48.00)
Exposure history, *n/N* (%)		68/78 (87.20)
Residents of Wuhan, *n/N* (%)		71/78 (91.03)
Menarche, median (IQR)		13 (12–13)
**Reproductive history**, ***n/N*** **(%)**		
Gravidity		2 (2–3)
Parity		1 (1–1.5)
Abortion times		1 (0–2)
**Medical history**, ***n/N*** **(%)**		
Comorbidities		17/78 (21.79)
Benign gynecological disease		9/75 (12.00)
Gynecological surgery history		27/75 (36.00)
**Symptoms on admission**, ***n/N*** **(%)**		
Fever		58/78 (74.36)
Cough		28/78 (35.90)
Sore throat		0/78 (0)
Fatigue		4/78 (5.13)
Myalgia		1/78 (1.28)
Dyspnea		8/78 (10.26)
Diarrhea		4/78 (5.13)
**Therapy**, ***n/N*** **(%)**		
Antiviral treatment		45/74 (60.81)
Antibiotic treatment		44/74 (59.46)

*Median (IQR): continuous variables were expressed as medians and interquartile ranges (IQR) as appropriate; n/N (%): categorical variables were summarized as the counts and percentages (%) in each category*.

### Menstruation Condition in COVID-19 Reproductive-Age Women

**Figure 1:**
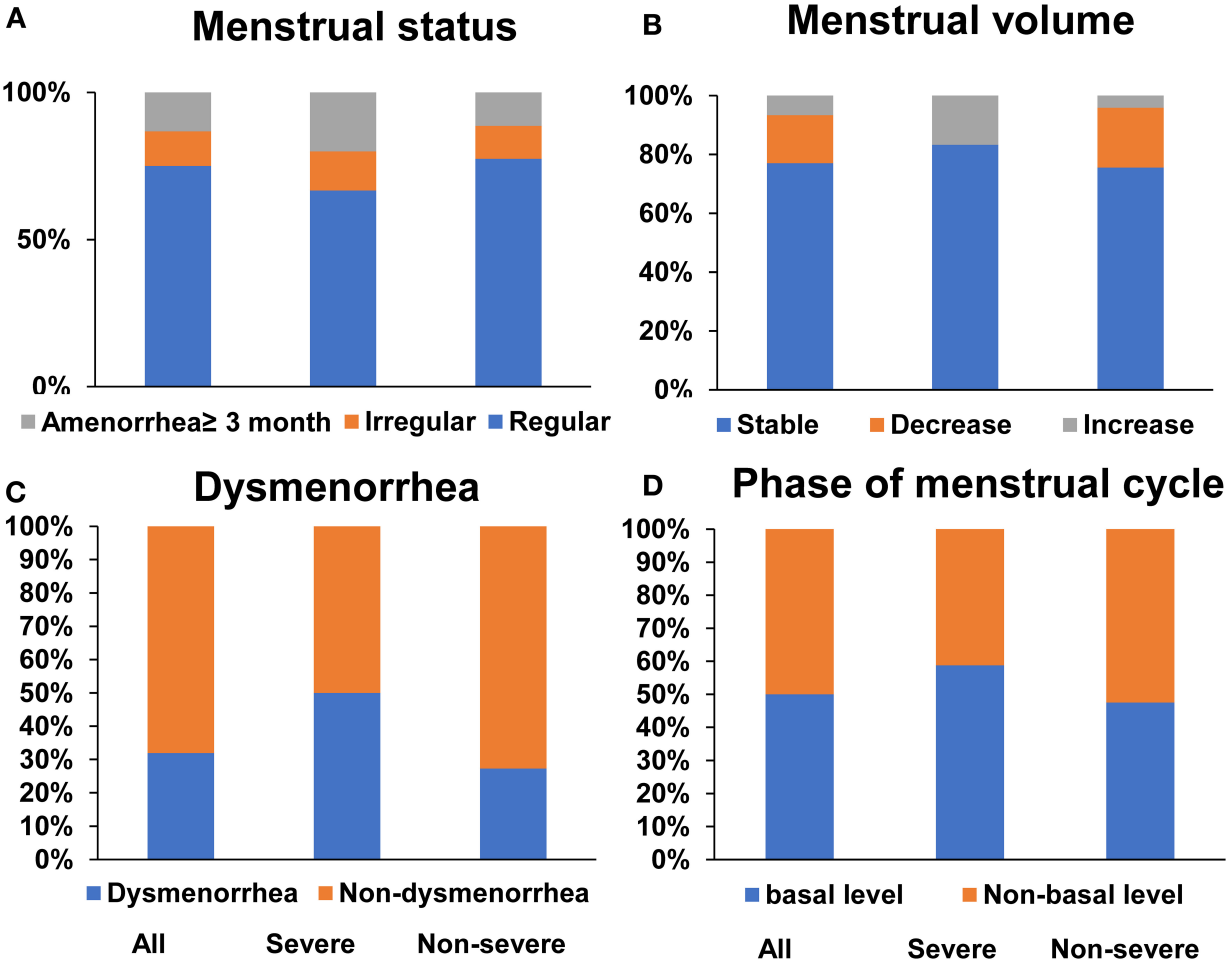
Menstruation condition in COVID-19 reproductive-age women. Menstrual status **(A)** and menstrual volumes **(B)** in last 3 months, dysmenorrhea history **(C)** and phase of menstrual cycle **(D)** in all, severe and non-severe COVID women, respectively.

Although 15% of the women infected with COVID-19 had an irregular menstrual cycle or amenorrhea. 51/68 (75%) still reported a regular menstrual cycle and 77% of them had a stable menstrual volume in the last 3 months. In addition, 31.9% COVID-19 women indicated a history of dysmenorrhea and half (39/78, 50%) were just in the basal level when blood was drawn. However, menstrual status (*P* = 0.55), menstrual volumes (*P* = 0.066), dysmenorrhea history (*P* = 0.12), and phase of menstrual cycle (*P* = 0.58) were similar without significant differences when comparison was made between severe and non-severe COVID-19 women (Figure 1).

### Comparison of Ovarian Reserve Tests and Female Sex Hormones Between COVID-19 Patients and Healthy Women

**Figure 2:**
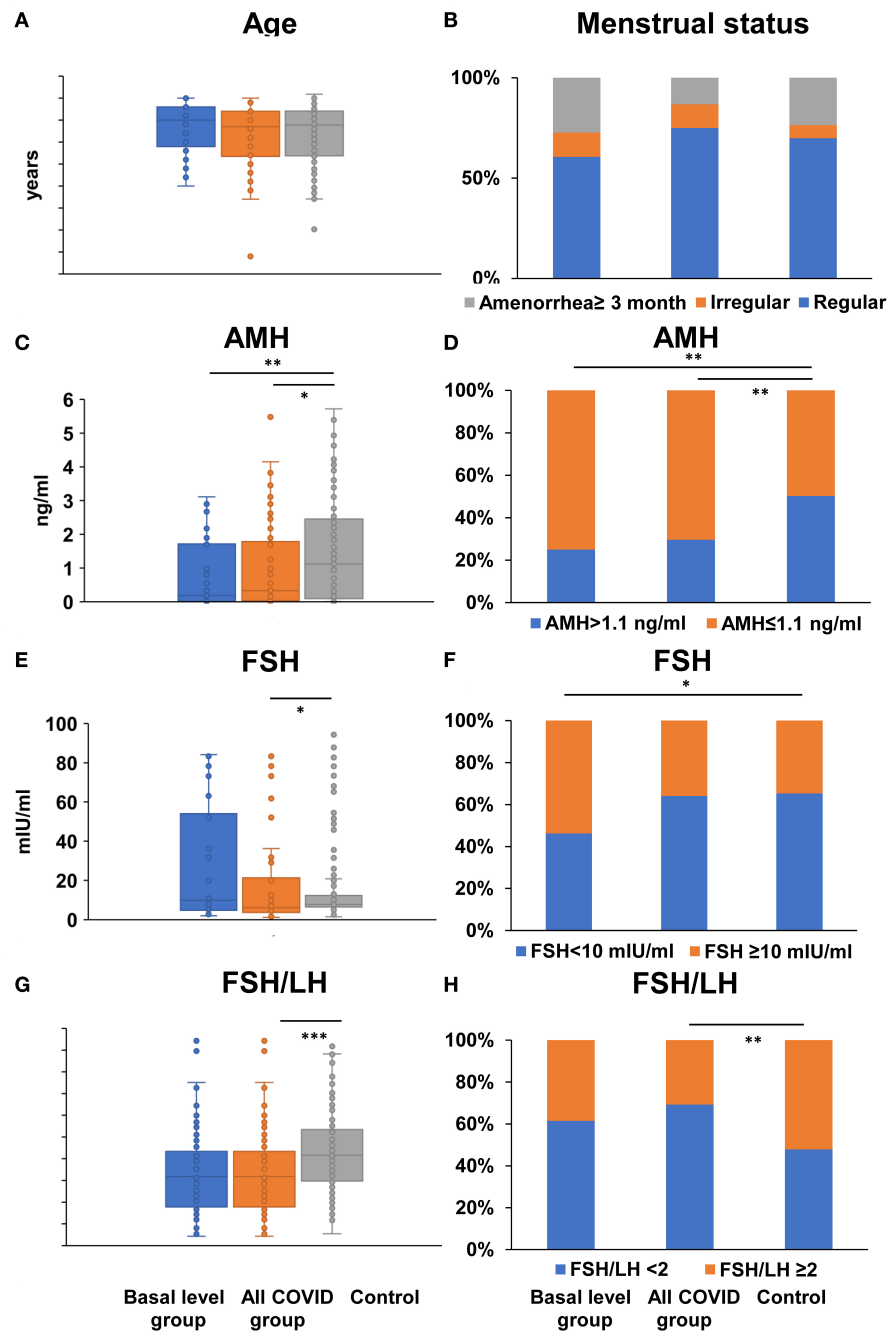
Comparison of age, menstrual status, and ovarian reserve tests between COVID-19 patients and healthy women. Differences of age **(A)**, menstrual status **(B)**, serum AMH **(C)**, FSH level **(E)**, the ratio of FSH to LH **(G)**, the proportion of AMH ≤ 1.1 ng/ml **(D)**, FSH ≥ 10 mIU/ml **(F)**, FSH/LH ≥ 2 **(H)** were compared between basal level/all COVID-19 group and healthy control women; *P < 0.05 **P < 0.01 ***P < 0.001.

Compared to the control group, there were no statistical differences regarding age and menstrual status in either basal level or the all- COVID-19 group of patients (Age: *P* = 0.213/0.776; Menstrual status: *P* = 0.462/0.138; Figures 2A,B, Supplementary Table 2), suggesting there is no differences in age and menstrual status between basal/all COVID-19 group and control, and the results were comparable. Both basal level and all COVID-19 women had significant lower serum AMH level (0.19/0.28 vs. 1.12 ng/ml, *P* = 0.003/0.027), and lower serum AMH ratio (AMH ≤ 1.1 ng/ml: 75.00/70.40 vs. 49.70%, *P* = 0.009/0.004) (Figures 2C,D, Supplementary Table 2). In addition, the serum FSH level was significantly different in comparisons between the all-COVID-19 group and healthy control (6.35 vs. 7.81 mIU/ml, *P* = 0.02); Despite no difference with basal level (*P* = 0.403), still more COVID-19 women had a relatively higher FSH (FSH ≥ 10 mIU/ml: 53.80 vs. 34.7%, *P* = 0.041) than in the basal level group. Although the lower ratio of FSH/LH (1.59 vs. 2.08, *P* < 0.001; FSH/LH ≥ 2: 30.80 vs. 52.19%, *P* = 0.003) was observed in both continuous and biological cutoff value in the all- COVID-19 group, these significant differences disappeared when compared with basal level and control groups (Figures 2E–H, Supplementary Table 2).

**Figure 3:**
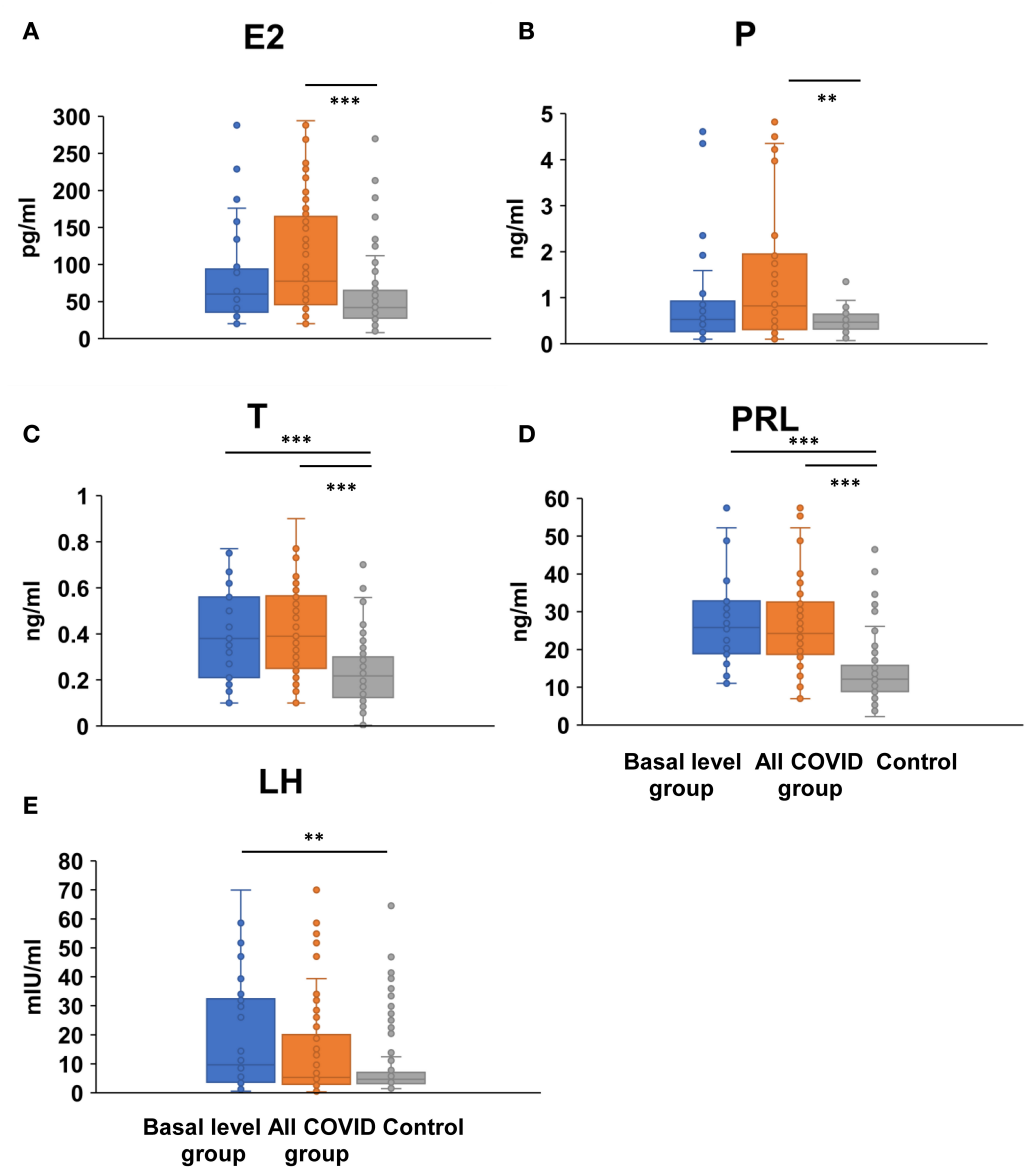
Comparison of female sex hormones between COVID-19 patients and healthy women. Differences of serum E2 **(A)**, P **(B)**, T **(C)**, PRL **(D)**, and LH levels **(E)** were compared between basal level/all COVID-19 group and healthy control women; **P < 0.01, ***P < 0.001.

The same lack of significant differences also occurred in serum E2 (54.00/72.50 vs. 41.91 pg/ml, *P* = 0.072, < 0.001) and *P* (0.53/0.77 vs. 0.47 ng/ml, *P* = 0.202/0.001) level from the all-COVID-19 to the basal level group when compared with control, suggesting an obvious fluctuate change within the menstrual cycle (Figures 3A,B, Supplementary Table 2). However, there were significant higher serum T (0.38/0.39 vs. 0.22 ng/ml, *P* < 0.001/ < 0.001) and PRL (25.43/24.10 vs. 12.12 ng/ml, *P* < 0.001/ < 0.001) levels in both basal and all-COVID-19 groups (Figures 3C,D, Supplementary Table 2). In addition, more COVID-19 women in the basal level were associated with higher LH (9.66 vs. 4.65 mIU/ml, *P* = 0.005) (Figure 3E, Supplementary Table 2).

### Hierarchical Linear Regression for Association of COVID-19 Disease With Ovarian Reserve

Results of the linear regression analyses with ovarian reserve were listed in Table 2. In univariate linear regression analysis, there is no correlation between ovarian reserve marker- serum AMH level and menarche (*P* = 0.48), BMI (*P* = 0.18), smoking (*P* = 0.94), benign gynecological disease (*P* = 0.49) and a gynecological operation history (*P* = 0.11) which may have potential influence on ovarian function. However, serum AMH level was negatively associated with age (β = −0.734, *P* < 0.001), COVID-19 disease (β = −0.162, *P* = 0.016), gravidity (β = −0.298, *P* < 0.001) and parity (β = −0.331, *P* < 0.001), respectively; and positively correlated with regular menstrual cycle (β = 0.427, *P* < 0.001). In multivariable hierarchical linear regression analyses, baseline characteristics including age, gravidity, and parity contributed to 51.4% of the variance in serum AMH level. After controlling for the baseline variables, regular menstrual cycle was positively associated with AMH, and accounted for 1.6% of the variance in AMH. COVID-19 disease was negatively related to AMH, which accounted for 3.2% of the decline in AMH. In all, after hierarchically adjusting for age and menstrual status, COVID-19 disease (β = −0.191; 95% CI: −1.177–0.327; *P* = 0.001) was significantly associated with serum AMH, suggesting an independent risk factor for ovarian reserve.

**Table 2:** Linear regression for association of COVID-19 disease with ovarian reserve AMH.

	**Univariate linear analysis**					
	**β**^**#**^		**95% CI**			***P*-value**
Age	−0.734		−0.212	−0.166		<0.001^***^
Menstrual status^※^	0.427		0.717	1.357		<0.001^***^
COVID-19	−0.162		−1.161	−0.121		0.016^*^
Gravidity	−0.298		−0.435	−0.168		<0.001^***^
Parity	−0.331		−1.069	−0.465		<0.001^***^
BMI	−0.1		−0.155	0.029		0.176
Menarche	−0.049		−0.222	0.105		0.481
Smoking	−0.002		−1.007	0.974		0.974
Benign gynecological disease	−0.054		−1.272	0.607		0.486
Gynecological operation history	−0.124		−1.127	0.109		0.106
**Variable**	**Multivariate hierarchical linear regression analysis**					
	**Step 1 (β)**	***P*****-value**	**Step 2 (β)**	***P*****-value**	**Step 3 (β)**	***P*****-value**
**Step 1**						
Age	−0.671	<0.001^***^	−0.606	<0.001^***^	−0.567	<0.001^***^
Gravidity	−0.095	0.171	−0.099	0.146	−0.116	0.081
Parity	−0.017	0.807	−0.006	0.925	0.045	0.506
**Step 2**						
Menstrual status^※^			0.145	0.017^*^	0.186	0.002^**^
**Step 3**						
COVID-19					−0.191	0.001^***^
F	58.560	<0.001^***^	5.793	0.017^*^	12.200	0.001^***^
R2	0.514		0.531		0.563	
ΔR2			0.016		0.032	

*Univariate and multivariate hierarchical linear regression models were used to assess the relationship between COVID-19 clinical characteristics and AMH; Only the variables showing statistical significance in univariate analysis were included in the multivariate hierarchical linear analysis*.

# *β = standardized regression coefficient*.

※ *Menstrual status including amenorrhea ≥3 months, irregular menstrual cycle, and regular menstrual cycle, as ordered rank variable*.

* P < 0.05

** P < 0.01

*** *P < 0.001*.

### Hierarchical Linear Regression for Association of COVID-19 Disease With Sex Hormones

Since serum T and PRL levels were significantly increased in both basal and all COVID-19 women, their associations with COVID-19 disease were further evaluated. Results of the linear regression analyses with sex hormones were listed in Tables 3, 4. In univariate linear regression analysis, dysmenorrhea (β = 0.252, *P* < 0.001), and COVID-19 disease (β = 0.448, *P* < 0.001) were positively associated with T. In multivariate hierarchical linear regression analyses, after controlling for dysmenorrhea, only COVID-19 disease (β = 0.411; 95% CI: 11.154–22.709; *P* < 0.001) was significantly associated with serum T, which accounted for 14.3% of the increase in T (Table 3).

**Table 3:** Linear regression for association of COVID-19 disease with sex hormone T.

	**Univariate linear analysis**			
	**β**^**#**^	**95% CI**		***P*-value**
Age	−0.120	−0.736	−0.043	0.081
Menstrual status^※^	0.075	−1.896	5.864	0.314
COVID-19	0.448	13.991	24.444	<0.001^***^
Gravidity	−0.040	−2.256	1.278	0.586
Parity	0.067	−2.179	6.057	0.354
BMI	−0.021	−1.134	0.854	0.781
Menarche	−0.130	−3.512	0.110	0.065
Dysmenorrhea	0.252	2.986	10.468	<0.001^***^
Smoke	−0.043	−14.530	7.604	0.538
**Variable**	**Multivariate hierarchical linear regression analysis**			
	**Step 1 (β)**	***P*****-value**	**Step 2 (β)**	***P*****-value**
**Step 1**				
Dysmenorrhea	0.252	<0.001^***^	0.092	0.198
**Step 2**				
COVID-19			0.411	<0.001^***^
F	12.585	<0.001^***^	33.424	<0.001^***^
R2	0.063		0.207	
ΔR2			0.143	

*Univariate and multivariate hierarchical linear regression models were used to assess the relationship between COVID-19 clinical characteristics and T; Only the variables showing statistical significance in univariate analysis were included in the multivariate hierarchical linear analysis*.

# *β = standardized regression coefficient*.

※ *Menstrual status including amenorrhea ≥3 months, irregular menstrual cycle, and regular menstrual cycle, as ordered rank variable*.

*** *P < 0.001*.

**Table 4:** Linear regression for association of COVID-19 disease with sex hormone PRL.

	**Univariate linear analysis**			
	**β**^**#**^	**95% CI**		***P*-value**
Age	−0.026	−0.346	−0.240	0.722
Menstrual status^※^	0.153	−0.084	5.18	0.058
COVID-19	0.473	9.984	17.497	<0.001^***^
Gravidity	0.001	−1.327	1.336	0.994
Parity	0.198	0.940	6.977	0.010^**^
BMI	−0.081	−0.832	0.287	0.338
Menarche	−0.092	−2.624	0.630	0.228
Dysmenorrhea	0.163	0.243	6.235	0.034^*^
Smoke	−0.090	−12.427	2.95	0.226
**Variable**	**Multivariate hierarchical linear regression analysis**			
	**Step 1 (β)**	***P*****-value**	**Step 2 (β)**	***P*****-value**
**Step 1**				
Dysmenorrhea	0.105	0.206	−0.036	0.636
Parity	0.183	0.028*	−0.078	0.299
**Step 2**				
COVID-19			0.497	<0.001^***^
F	4.424	0.014^*^	41.902	<0.001^***^
R2	0.056		0.263	
ΔR2			0.207	

*Univariate and multivariate hierarchical linear regression models were used to assess the relationship between COVID-19 clinical characteristics and PRL; Only the variables showing statistical significance in univariate analysis were included in the multivariate hierarchical linear analysis*.

# *β = standardized regression coefficient*.

※ *Menstrual status including amenorrhea ≥3 months, irregular menstrual cycle and regular menstrual cycle, as ordered rank variable*.

* P < 0.05

** P < 0.01

*** *P < 0.001*.

Similarly, dysmenorrhea (β = 0.163, *P* = 0.034), parity (β = 0.198, P = 0.010) and COVID-19 disease (β = 0.473, *P* < 0.001) were associated with PRL in univariate linear regression. When controlled with dysmenorrhea and parity in multivariate hierarchical linear regression, only COVID-19 disease (β = 0.497; 95% CI: 10.787-2–0.266; *P* < 0.001) was significantly associated with serum PRL, which accounted for 20.7% of the increase in PRL (Table 4).

### Laboratory Characteristics of COVID-19 Women and Their Associations With Ovarian Reserve and Sex Hormones

The laboratory characteristics regarding immunity and inflammation are listed in Table 5. Their associations with ovarian reserve and sex hormones using Pearson correlation indicated that ferritin (*P* = 0.042) and Immunoglobulin M (*P* = 0.029) were positively associated with T; Complement 4 (*P* = 0.043) was negatively associated with T. No significant correlations between these laboratory characteristics and AMH or PRL were observed.

**Table 5:** The laboratory characteristics of COVID-19 women and their associations with ovarian reserve and sex hormones.

	**Median, IQR**	**AMH (ng/ml)**			**T (ng/ml)**			**PRL (ng/ml)**		
		***N***	**R**	***P*-value**	***N***	**R**	***P*-value**	***N***	**R**	***P*-value**
**Age**										
**Laboratory characteristics**										
White blood cell count (WBC, ×10^9^/L)	5.23 (4.3–7.27)	67	0.102	0.413	70	0.007	0.952	70	−0.183	0.13
Lymphocyte count (LC, x10^9^/L)	1.42 (1.07–1.83)	67	0.162	0.191	70	−0.058	0.636	70	−0.088	0.466
Erythrocyte sedimentation rate (ESR, mm/h)	20.5 (8.5–37)	37	−0.137	0.42	38	−0.144	0.387	38	−0.01	0.955
C-reactive protein (CRP, mg/L)	1.75 (0.55–14.25)	58	−0.216	0.104	60	0.166	0.206	60	−0.139	0.289
Procalcitonin (PCT, pg/ml)	0.03 (0.02–0.06)	49	−0.096	0.513	52	0.267	0.056	52	−0.079	0.578
Ferritin(μg/L)	139.9 (78.9–309.2)	27	−0.187	0.349	27	0.394	0.042^*^	27	−0.289	0.143
Immunoglobulin A (IgA, g/L)	2.2 (1.89–2.75)	14	−0.299	0.299	14	0.22	0.45	14	0.158	0.59
Immunoglobulin G (IgG, g/L)	13 (10.8–15.8)	14	0.17	0.562	14	−0.027	0.928	14	0.347	0.224
Immunoglobulin M (IgM, g/L)	1.23 (0.73–1.76)	14	−0.369	0.194	14	0.583	0.029^*^	14	−0.33	0.249
Complement 3 (C3, g/L)	0.87 (0.71–1.02)	14	−0.519	0.057	14	−0.188	0.521	14	−0.058	0.843
Complement 4 (C4, g/L)	0.2 (0.15–0.26)	14	0.029	0.92	14	−0.547	0.043^*^	14	−0.323	0.26
Interleukin 10 (IL10, pg/ml)	5 (5–5)	48	−0.046	0.756	51	0.034	0.814	51	−0.162	0.255
Interleukin 1β (IL1β, pg/ml)	5 (5–5)	48	−0.078	0.6	52	−0.117	0.41	52	−0.093	0.511
Interleukin 2R, (IL2R, U/ml)	431 (281.5–612)	49	−0.196	0.178	53	−0.041	0.772	53	0.006	0.968
Interleukin 6 (IL6, pg/ml)	2.47 (1.5–7.94)	49	−0.139	0.34	53	0.213	0.127	53	−0.263	0.057
Interleukin 8 (IL8, pg/ml)	9 (5.75–20.9)	49	−0.203	0.162	53	0.213	0.126	53	0.038	0.79
Tumor necrosis factor α (TNFα, pg/ml)	7.7 (4.9–9.15)	49	−0.18	0.217	53	0.019	0.891	53	−0.08	0.571

*Pearson correlation analyses were used with laboratory characteristics and ovarian reserve/sex hormones*.

* *P < 0.05*.

## Discussion

It is not yet clear what effects, if any, COVID-19 will have on female reproductive function and pituitary gonadal axis. In this study we analyzed the menstrual condition in reproductive aged women infected by COVID-19 and compared the ovarian reserve markers and sex hormone profiles of COVID-19 patients with age-matched healthy women. AMH is considered the earliest and most sensitive ovarian reserve marker, which allows for testing anytime throughout the cycles (4). Our results indicate that although no obvious menstrual cycle change was observed, women affected by COVID-19 have a significantly lower serum AMH level and higher T/PRL level, suggesting a poor ovarian reserve and abnormal reproductive hormones compared to the age-matched healthy unaffected women. Hierarchical linear regression showed that COVID-19 disease was likely to be an independent risk factor respecting ovarian function as represented by AMH, T, and PRL levels, even after adjusted for age, menstrual cycle, and parity variations. Therefore, COVID-19 was assumed to have a potential deleterious effect on ovarian reserve and endocrine function.

This observed ovarian injury may be caused directly by coronavirus binding to the *ACE2* receptor and entering the cell through *TMPRSS2*, leading to a cytopathic effect mediated by local replication of the SARS-CoV-2. The available evidence suggests that *ACE2* is widely expressed in the ovary, uterus, vagina, and placenta. *ACE2* can be detected in ovaries from humans (15–18) to animals (19), and our data also verify the expression of *ACE2* and *TMPRSS2* in the human ovary (Not published). Co-expression of *ACE2* and *TMPRSS2* was observed both in oocytes and ovarian granulosa cells, concerning a possible effect of SARS-CoV-2 on female reproduction. Furthermore, some transcriptomic data indicates that ACE2 is also expressed in human cumulus cells (23). Meanwhile, Ang II and Ang-(1–7), which are modulated by ACE2, can induce steroid secretion, facilitate follicle development and atresia, regulate oocyte maturation, and influence ovulation. Taking these functions into account, SARS-CoV-2 may disturb the female reproductive function through ACE2 (19). However, current data suggests that the female reproductive system may be spared from viral infection (17, 24). Our data also suggested that no virus was detected in the lower genital tract either, including vaginal fluid, and cervical exfoliated cells (7). This is in accordance with the insufficient evidence for vertical disease transmission from parents to children (5, 6). More research is needed to verify existence of the virus in the female reproductive system, especially in the ovary.

However, more evidence is emerging to indicate that a virus attack was not the only way to impair organ function. The impairment can be as an indirect result of systemic responses to respiratory failure, or the harmful immune response induced by viral infection (24). Therefore, we also analyzed the relationship between serum AMH/T/PRL and the immune and inflammatory characteristics of COVID-19 patients in order to determine the impacts of the corresponding “immune/inflammatory storm” on ovarian function. However, only T had significant differences with several laboratory characteristics, but not AMH or PRL. A larger cohort would help us to better determine the association between immunity/inflammation and the ovarian function.

Since there is no obvious statistical correlation of cytokines with AMH and PRL level, there might be possible reasons other than immunity or inflammation that lead to ovarian damage; there will need further study. Notably, the serum pituitary hormone LH and the PRL level were significantly elevated in COVID-19 patients, indicating a severe endocrine disorder. Studies reported that the nervous system could be damaged by SARS-CoV-2 (10) and that SARS-CoV can be found in the pituitary gland (24). This hormone elevation might partly result from the direct impact on brain tissue. On the other hand, women easily become anxious during the pandemic, which is also a risk for endocrine disorder (25). In our study, thirty-six (36) of 75 (48.0%) patients described a recent mental disorder such as anxiety, depression, or insomnia which could cause high PRL and dysfunction of hypothalamus-pituitary-ovary (HPO) axis. Meanwhile, the elevated LH would stimulate theca cells to secrete more T, probably causing a secondary ovulation dysfunction at a later time. This situation might be even worse for the polycystic ovary syndrome in women who have already had an endocrine problem (26). Unlike serum AMH and T level, the other ovarian hormones, serum E2, and serum P, were not significantly different in the basal COVID-19 group and the control group. Taken together, we infer that the elevated LH and PRL are more likely to be caused by nervous system injuries and pituitary dysfunction, while decreased AMH and elevated T probably result from ovarian dysfunction, such as the damage of granulosa and theca cells. The deleterious effects of COVID-19 disease possibly involved the direct damage of ovarian follicles, virus-induced hyperinflammatory, or postinfectious immune mediated processes, abnormal sex-hormone secretion, and dysfunction of HPO axis process. More evidence is needed to validate the foregoing hypothesis.

Although there have been a series of changes in reproductive hormones, life history theory suggests that rates of female reproductive aging and the timing of major reproductive events are shaped based on the sensitivity of morbidity-mortality to the organism's resource-allocation decisions (27). In a body system with finite resources, investments in reproduction are proposed to result in trade-offs (28), in order to maintain enough energy in vital organs to survive in a COVID-19 pandemic. For example, a study reported that the desire for pregnancy decreased during this period (29). Therefore, the reproductive function might be first sacrificed to retain the basic capacity to maintain life in the short term. Later it would be recovered when the body's condition is improved and could permit reproduction.

This study has several limitations. First, regression analysis showed that while ovarian reserve marker AMH and sex hormone T/PRL are significantly correlated with COVID-19 disease, the causal relationship remains unclear. Second, the hormone levels were determined based on only one blood sample per patient regardless of the phase of menstrual cycle, and within only a short time of infection by SARS-CoV-2. Third, no existence of SARS-CoV-2 in ovary was detected, especially in oocyte, which is more straightforward evidence for ovarian injury caused by SARS-CoV-2. Fourth, the limited sample size of serum sex hormones might not be large enough, which may affect the power of statistical analysis. Fifth, the population in our study includes some older perimenopausal women who probably have fluctuating sex hormone levels that leads to data bias. More samples from younger women and long-term prospective cohorts are needed to further determine the effects of COVID-19 diseases on ovarian function.

## Conclusions

This study provides the initial clinical evidence showing that female COVID-19 patients probably have an ovarian injury, with a poor ovarian reserve of decreased AMH and reproductive endocrine disorder of aberrant sex hormone levels, especially high T and PRL. The results inferred a potential diminished ovarian reserve and reduced reproductive potential in a short time. Direct virus attack, excessive immune, or inflammatory response and dysfunction of HPO axis may all contribute to the abnormality of ovarian function under COVID-19, finally leading to ovarian injury. However, more evidence, including both epidemiologic and animal studies, are needed to verify the impact of coronavirus on the ovary. Further studies are warranted of the reproductive involvement of coronavirus infections, particularly regarding recovered patients in a larger cohort and long-term follow-up, paying more attention to ovarian function evaluation, fertility outcome, and endocrine condition among patients recovered from SARS-CoV-2 infection after this pandemic, especially in reproductive-aged women.

## Data Availability Statement

The original contributions presented in the study are included in the article/Supplementary Material, further inquiries can be directed to the corresponding author/s.

## Ethics Statement

The studies involving human participants were reviewed and approved by Medical Ethical Committee of Tongji Hospital of Huazhong University of Science and Technology (TJ-IRB20200214). Written informed consent for participation was not required for this study in accordance with the national legislation and the institutional requirements.

## Author Contributions

TD was responsible for study concept and design, analysis and interpretation of data, and drafting of the manuscript. TW, JZ, PC, ZC, SZ, SY, and WM were responsible for data acquisition and analysis. MZ, YR, JC, and XPM was responsible for acquisition, analysis of data, and critical revision of the manuscript. XYM and SW were responsible for the study concept and design, critical revision of the manuscript, and study supervision. All authors contributed to the article and approved the submitted version.

## Conflict of Interest

The authors declare that the research was conducted in the absence of any commercial or financial relationships that could be construed as a potential conflict of interest.
