# Supplementary material for: Analysis of Ovarian Injury Associated With COVID-19 Disease in Reproductive-Aged Women in Wuhan, China: An Observational Study
Source: Front Med (Lausanne). 2021 Mar 19;8:635255. doi: 10.3389/fmed.2021.635255 (PMC8017139; doi:10.3389/fmed.2021.635255)
Supplement: Supplementary file 1 [file Table_1.doc]

**Supplementary Table 1 Comparison of ovarian reserve tests and female sex hormones in all COVID-19 patients between with and without comorbidities, benign gynecological disease or gynecological surgery history.**

|  | Medical history | | | | | | | | |
| --- | --- | --- | --- | --- | --- | --- | --- | --- | --- |
|  | Comorbidities | Without comorbidities | *P value* | Benign gynecological disease | Without benign gynecological disease | *P value* | Gynecological surgery history | Without gynecological surgery history | *P value* |
| n/N (%) | 17/78 (21.79%) | 61/78 (78.21%) |  | 9/75 (12%) | 66/75 (88%) |  | 27/75 (36%) | 48/75 (64%) |  |
| AMH |  |  |  |  |  |  |  |  |  |
| Median (IQR), ng/ml | 0.33 (0.01-1.76) | 0.25 (0.03-1.80) | 0.960 | 0.14 (0.01-1.30) | 0.27 (0.03-1.83) | 0.312 | 0.20 (0.03-1.52) | 0.49 (0.02-1.81) | 0.624 |
| FSH |  |  |  |  |  |  |  |  |  |
| Median (IQR), mIU/ml | 5.81(3.22-14.06) | 6.53 (3.78-30.41) | 0.442 | 4.62 (3.31-53.65) | 6.56 (3.83-23.42) | 0.660 | 6.72 (4.64-17.31) | 6.11 (3.58-31.08) | 0.783 |
| FSH/LH ratio |  |  |  |  |  |  |  |  |  |
| Median (IQR) | 1.32 (1.01-2.08) | 1.59 (0.85-2.34) | 0.880 | 1.59 (0.75-1.82) | 1.63 (0.92-2.45) | 0.546 | 1.31 (0.82-2.72) | 1.66 (0.96-2.24) | 0.529 |
| E2 |  |  |  |  |  |  |  |  |  |
| Median (IQR), pg/ml | 75.00 (34.50-177.00) | 70.00(42.00-162.00) | 0.832 | 60.00 (42.00-155.00) | 70.00(40.75-165.75) | 0.744 | 88.00 (46.00-195.00) | 69.00(40.25-163.50) | 0.490 |
| P |  |  |  |  |  |  |  |  |  |
| Median (IQR), ng/ml | 0.53 (0.26-4.06) | 0.85(0.34-1.94) | 0.637 | 0.51 (0.30-4.24) | 0.83 (0.31-1.91) | 0.935 | 0.51 (0.26-1.09) | 0.91 (0.38-2.25) | 0.071 |
| T |  |  |  |  |  |  |  |  |  |
| Median (IQR), ng/ml | 0.43 (0.34-0.55) | 0.37 (0.22-0.57) | 0.553 | 0.43 (0.30-0.62) | 0.39 (0.22-0.56) | 0.448 | 0.39 (0.27-0.57) | 0.39 (0.25-0.57) | 0.724 |
| LH |  |  |  |  |  |  |  |  |  |
| Median (IQR), mIU/ml | 4.71 (2.73-11.24) | 5.83 (3.11-24.41) | 0.414 | 3.40 (2.06-34.08) | 5.68 (3.31-19.74) | 0.922 | 5.09 (3.96-14.35) | 5.72 (2.20-21.18) | 0.921 |
| PRL |  |  |  |  |  |  |  |  |  |
| Median (IQR), ng/ml | 33.04 (19.26-48.81) | 23.52 (18.16-29.01) | 0.062 | 24.31 (15.41-35.44) | 24.10 (18.75-32.82) | 0.942 | 23.23 (16.17-28.84) | 24.85 (18.73-34.42) | 0.547 |

Median (IQR): continuous variables were expressed as medians and interquartile ranges (IQR) as appropriate; n/N (%): categorical variables were summarized as the counts and percentages (%) in each category

Mann-Whitney U tests (nonparametric)were applied to continuous variables, chi-square tests and Fisher’s exact tests were used for categorical variables as appropriate.

*P<0.05 **P<0.01 ***P<0.001

Comorbidities included hypertension, diabetes, cardiovascular disease etc.

Benign gynecological diseases histories included vaginitis, pelvic inflammatory disease, fibroids etc.

Gynecological surgery histories included cesarean, artificial abortion, tubal surgery etc.
