# Supplementary material for: Analysis of Ovarian Injury Associated With COVID-19 Disease in Reproductive-Aged Women in Wuhan, China: An Observational Study
Source: Front Med (Lausanne). 2021 Mar 19;8:635255. doi: 10.3389/fmed.2021.635255 (PMC8017139; doi:10.3389/fmed.2021.635255)
Supplement: Supplementary file 2 [file Table_2.doc]

**Supplementary Table 2 Comparison of ovarian reserve tests and female sex hormones between COVID-19 patients and healthy women**

| **Hormones** | **Female COVID-19 patients** | | **Age matched healthy women** | *P* value  (Basal vs Control) | *P* value  (All vs Control) |
| --- | --- | --- | --- | --- | --- |
|  | Basal level group | All COVID-19 group | Control |
|  | (N=39) | (N=78) | (N=151) |
| Age, Median (IQR) | 45.00 (39.00-48.00) | 43.50 (36.75-47.00) | 43.90 (36.90-47.07) | 0.213 | 0.776 |
| Menstrual status, n/N (%) |  |  |  |  |  |
| Regular | 20/33(60.60) | 51/68 (75.00) | 85/123 (69.90) |  |  |
| Irregular | 4/33 (12.10) | 8/68 (11.80) | 8/123 (6.50) |  |  |
| Amenorrhea≥ 3 month | 9/33 (27.30) | 9/68 (13.20) | 29/123 (23.60) | 0.462 | 0.138 |
| AMH |  |  |  |  |  |
| Median (IQR), ng/ml | 0.19 (0.01-1.52) | 0.28 (0.03-1.76) | 1.12 (0.09-2.46) | 0.003^**^ | 0.027^*^ |
| AMH≤1.1 ng/ml, n/N (%) | 27/36 (75.00) | 50/71 (70.40) | 75/151 (49.70) | 0.009^**^ | 0.004^**^ |
| FSH |  |  |  |  |  |
| Median (IQR), mIU/ml | 12.54 (5.01-61.88) | 6.35 (3.73-23.42) | 7.81 (6.47-12.32) | 0.403 | 0.020^*^ |
| FSH ≥10 mIU/ml, n/N (%) | 21/39 (53.80) | 28/78 (35.90) | 52/150 (34.70) | 0.041^*^ | 0.884 |
| FSH/LH ratio |  |  |  |  |  |
| Median (IQR) | 1.83 (1.02-2.41) | 1.59 (0.89-2.17) | 2.08 (1.49-2.67) | 0.059 | <0.001^***^ |
| FSH/LH ≥2, n/N (%) | 15/39 (38.50) | 24/78 (30.80) | 74/142 (52.19) | 0.150 | 0.003^**^ |
| E2 |  |  |  |  |  |
| Median (IQR), pg/ml | 54.00 (35.00-91.00) | 72.50(42.50-165.00) | 41.91 (27.64-65.17) | 0.072 | <0.001^***^ |
| P |  |  |  |  |  |
| Median (IQR), ng/ml | 0.53 (0.26-0.92) | 0.77 (0.31-1.93) | 0.47 (0.32-0.65) | 0.202 | =0.001^**^ |
| T |  |  |  |  |  |
| Median (IQR), ng/ml | 0.38 (0.21-0.56) | 0.39 (0.24-0.56) | 0.22 (0.12-0.30) | <0.001^***^ | <0.001^***^ |
| LH |  |  |  |  |  |
| Median (IQR), mIU/ml | 9.66 (3.76-31.89) | 5.34 (3.07-21.06) | 4.65 (3.14-7.02) | 0.005^**^ | 0.193 |
| PRL |  |  |  |  |  |
| Median (IQR), ng/ml | 25.43 (18.82-32.75) | 24.10 (18.59-32.29) | 12.12 (8.86-15.81) | <0.001^***^ | <0.001^***^ |

Median (IQR): continuous variables were expressed as medians and interquartile ranges (IQR) as appropriate; n/N (%): categorical variables were summarized as the counts and percentages (%) in each category

Mann-Whitney U tests (nonparametric)were applied to continuous variables, chi-square tests and Fisher’s exact tests were used for categorical variables as appropriate.

^*^P<0.05 ^**^P<0.01 ^***^P<0.001
